# Functional subgroups of rat and human sensory neurons: a systematic review of electrophysiological properties

**DOI:** 10.1007/s00424-021-02656-6

**Published:** 2022-01-15

**Authors:** Jannis Körner, Angelika Lampert

**Affiliations:** 1 Institute of Physiology, Uniklinik RWTH Aachen, Pauwelsstrasse 30, 52074 Aachen, Germany; 2 Clinic of Anesthesiology, Uniklinik RWTH Aachen, Pauwelsstrasse 30, 52074 Aachen, Germany

## Abstract

Sensory neurons are responsible for the generation and transmission of nociceptive signals from the periphery to the central nervous system. They encompass a broadly heterogeneous population of highly specialized neurons. The understanding of the molecular choreography of individual subpopulations is essential to understand physiological and pathological pain states. Recently, it became evident that species differences limit transferability of research findings between human and rodents in pain research. Thus, it is necessary to systematically compare and categorize the electrophysiological data gained from human and rodent dorsal root ganglia neurons (DRGs). In this systematic review, we condense the available electrophysiological data defining subidentities in human and rat DRGs. A systematic search on PUBMED yielded 30 studies on rat and 3 studies on human sensory neurons. Defined outcome parameters included current clamp, voltage clamp, cell morphology, pharmacological readouts, and immune reactivity parameters. We compare evidence gathered for outcome markers to define subgroups, offer electrophysiological parameters for the definition of neuronal subtypes, and give a framework for the transferability of electrophysiological findings between species. A semiquantitative analysis revealed that for rat DRGs, there is an overarching consensus between studies that C-fiber linked sensory neurons display a lower action potential threshold, higher input resistance, a larger action potential overshoot, and a longer afterhyperpolarization duration compared to other sensory neurons. They are also more likely to display an infliction point in the falling phase of the action potential. This systematic review points out the need of more electrophysiological studies on human sensory neurons.

## Introduction

Sensory neurons are the cellular functional units in pain signal generation and transmission. Their cell bodies are located in the dorsal root ganglia (DRG) or trigeminal ganglia (TG). Yet the cellular composition of DRGs encompasses a broadly heterogeneous population of highly specialized neurons. Due to their high pathophysiological relevance, it is of significant interest to identify functional subgroups and link them to their function in the body. Even more challenging, but severely needed, is the identification of reliable biomarker for each subgroups (molecular or functional), which would substantially foster our understanding of underlying disease mechanisms, e.g., for neuropathic pain, and the development of specific treatments [11].

Traditionally, sensory neurons have been classified according to their fiber conduction velocity (CV) and degree of myelination as either fast conduction myelinated A-fibers, intermediate conducting, thinly myelinated Aδ-fibers, or slowly conducting unmyelinated C-fibers as reviewed in Middleton et al. [24]. Nociceptors respond to noxious stimuli and are mainly C-fibers and Aδ-fibers, but also some Aβ-fibers can be classified as so-called high threshold mechanoceptors (HTMs). C-fibers can be further subdivided in those which react to mechanical stimuli (C_M_-fibers) and those which do not (C_M_i-fibers). The latter are also called silent or sleeping nociceptors, which can be recruited after sensitization, e.g., in inflammatory states, and are involved in neuropathic pain states [24]. Voltage-gated sodium currents are the basis of the fast upstroke of the action potential (AP) and thus crucial for determining cellular excitability. They are traditionally classified due to their sensitivity to tetrodotoxin (TTX) into resistant (TTXr) and sensitive (TTXs), and are studied intensely in sensory neurons.

To date, several methodological approaches have been used to study and further classify the heterogeneity in sensory neurons including microneurography [24] and single cell RNA sequencing approaches [20, 40]. Besides recent breakthroughs in single cell RNASeq technologies leading to profound insights in the molecular choreography enabling somatosensation and nociception, large efforts have been made to also functionally characterize neuronal subgroups with patch-clamp and sharp electrode approaches. While both mouse and rats have been extensively studied with those approaches, most studies focusing on the characterization of neuronal subgroups were performed on rat DRG tissue. For this reason, we focus on the comparison between rat and human sensory neurons in this review.

Lately, it has become clear that there are substantial differences in the nociceptive system of rodents and human [32]. Recent drawbacks in the establishment of new pain therapies are partly interpreted as a translational gap between animal and human studies [16]. As a consequence, the focus in the development of new pain therapies is moving towards the use of human or human-like biological models [12, 31]. Thus, a functional electrophysiological characterization of subtypes in sensory neurons also in those models is needed as one part of the description of the nociceptive system in humans.

In the last 40 years, a large body of literature aiming to subclassify rat DRG tissue accumulated. Yet, those studies use rather diverse electrophysiological methods, tissue preparations, and most importantly they vary greatly in their selection of electrical features and subgroups they were comparing. For electrophysiology on human DRG tissue, there are only few studies published and therefore the knowledge is more limited.

To reach the objective of a structured electrophysiological characterization of neuronal subtypes in human primary sensory neurons, we believe it is necessary to summarize the existing current knowledge on electrophysiological subcategories in sensory neurons both of rats and humans. Here, we set out to give a structured overview of the published electrophysiological characterizations and classifications of primary sensory neurons of the two species. The scope of this review is to (1) condense these findings in a systematical manner and (2) offer an assessment on the transferability of electrophysiological knowledge from one species to the other. We present a structured overview on subgroups in those neuronal populations that have been studied with electrophysiological approaches in rats and humans and compare the outcomes (characterizing electrophysiological parameters) to offer a framework of future studies investigating sensory neuron subgroups in human or human-like biological systems.

## Methods

### Literature search

The study was performed in accordance with the Preferred Reporting Items for Systematic Reviews and Meta-Analysis (PRISMA) statement [27]. The study followed a review protocol which was not published before. Advanced literature search was performed using the PUBMED databases with the search string: ((“dorsal root ganglion”) OR (“Sensory neurone”) OR (“sensory neuron”)) AND ((nociceptor) OR (“c-fibres”) OR (“c-fibre”) OR (“c-fiber”) OR (“c-fibers”) OR (“c-cells”) OR (“pain”)) AND ((“action potential”) OR (“whole cell patch-clamp”)) AND ((“1900”[Date—Publication]: “3000”[Date—Publication])). The Date was limited between 01 January 1900 and 02 August 2021. Only peer-reviewed studies published in English were considered eligible for the systematic review. Due to the large number of potential studies, an initial screening via title and abstract was conducted to remove papers that were not suitable for the scope of the review (see “Eligibility criteria” below). This selection was performed by J. K. After screening, all full text articles were obtained.

### Eligibility criteria

**Fig. 1:**
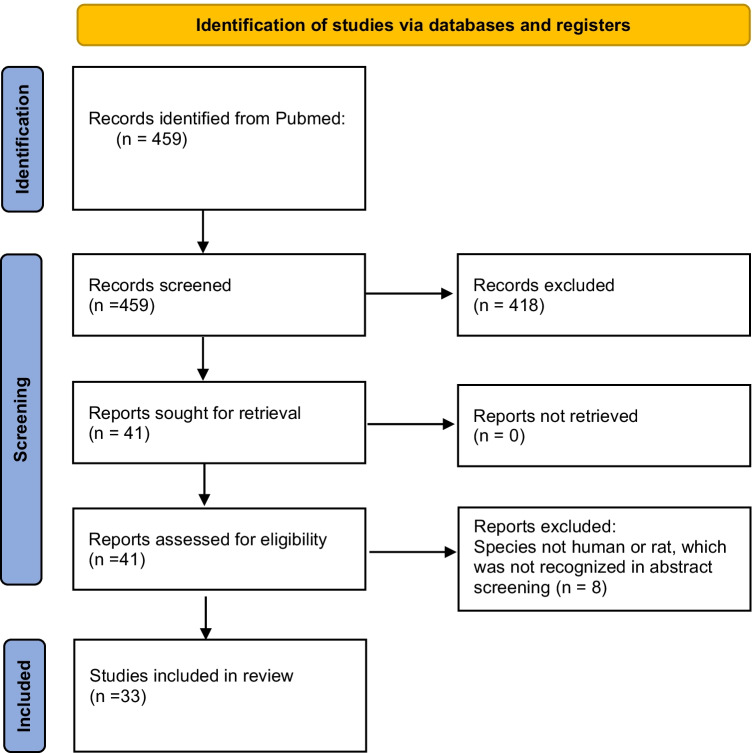
Flowchart of systematic literature research. PRISMA flowchart of the presented study adapted from Page et al. [27]

The studies had to fulfill the following criteria: (1) experimental studies on rat or human DRG tissue (2) comparison of electrophysiological cell features between subgroups of neurons in healthy tissue as defined in (1). Electrophysiological cell features are defined as results from single cell electrical recordings via whole-cell patch clamp, perforated patch, or sharp electrodes (microelectrodes) in voltage- or current-clamp mode. Assessment of immunostainings (or leptin binding) and cell morphology were also included. We considered all of the following tissue preparations: intact DRGs ex vivo, in vivo, dissociated DRGs, and whole mount DRG preparation. We did not distinguish the studies based on the recording method nor the tissue preparation used. Initially, 459 publications were screened. After application of the inclusion criteria, 30 studies investigating rat DRG tissue and 3 studies studying human DRG tissue were included (Fig. 1).

### Data extraction, synthesis, and assessment

**Fig. 2:**
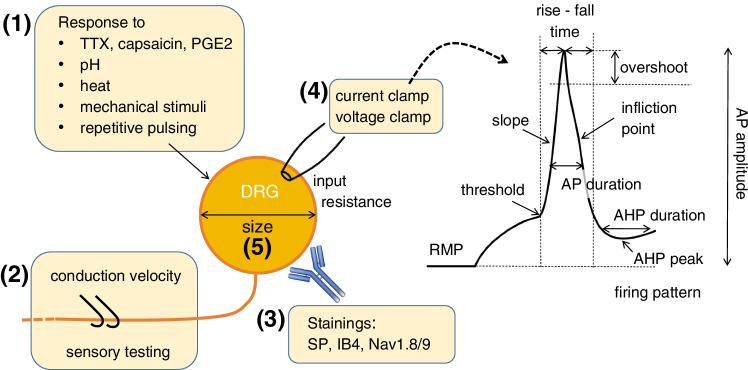
Illustration of extracted outcome parameters. Overview of the parameters collected from the included studies. The data extracted from the included literature consist of: (1) response of neurons to various stimuli including pharmacological approaches (TTX, capsaicin, PGE2), environmental changes of pH and temperature, as well as mechanical stimulation or repetitive electrical stimulation. (2) Assessment of properties of the somatic afferents including axon conduction velocity and sensory testing in the neurons receptive field. (3) Immunofluorescence data with stainings including SP, IB4, and Nav1.8/1.9 (down middle). (4) Electrophysiological parameters in both voltage clamp and current clamp mode as depicted in a schematic action potential. (5) Soma diameter as a measure of cell morphology

The data extraction was performed by JK. The data included the species, the number of cells included into the study, any restriction on selection of cells included (e.g., only small diameter DRGs), electrophysiological method, the method of tissue preparation, the way the study performed subcategorization of neurons, and the electrophysiological parameters assessed (see Fig. 2, Tables 1, and 2).

**Table 1:** Summary of studies and extracted data for rat DRGs. “Groups determination” indicates whether data were grouped post or pre hoc. *RMP*, resting membrane potential; *AP*, action potential; *APD*, action potential duration; *capsaicin*, capsaicin sensitive; *AHP*, afterhyperpolarization; *TTXs/r*, tetrodotoxin resistant/sensitive; *RT*, rising time; *FT*, falling time; *TTP*, time to peak. In cases where CV or size as subgrouping parameter is not quantified, two parameters, e.g., CV + APD, were used for categorization

	Cells included	Method	n Neurons	Preparation	Subgrouping into	Assessed parameters	Groups determination
[14]	All	Intracellular microelectrode	70	Intact DRG ex vivo	CV: C < 1.4 m/s; Aδ 2.2–8 m/s, Aβ 14–30 m/s, Aα 30–55 m/s, and infliction point	RMP, APAmp, APD, AHP-duration, input resistance, AHP peak, infliction point, dep. slope, overshoot, repetitive stimulation	Unclear
[43]	All	Intracellular microelectrode	211	Intact DRG ex vivo	CV: C < 1.3 m/s; Aδ 1.3–12 m/s, Aα/Aβ > 12 m/s, and infliction point	RMP, threshold, APAmp, APD, AHP-duration, AHP peak, infliction point, overshoot, RT, FT, repetitive stimulation, firing pattern, TTXs	Pre hoc
[8–10]	All	Whole cell and perforated patch	246	Dissociated DRGs	(1) Cell size small 20–27 µm, medium 33–37 µm, large 41–48 µm (2) SP + / − (3) Infliction point + / −	RMP, infliction point, size, capsaicin, PGE2s	Unclear
[8]	All	Whole cell and perforated patch	258	Dissociated DRGs	(1) PGE2 treatment + / − (2) AHP slow + / − neurons	Infliction point, size, capsaicin, PGE2s	Pre hoc
[9]	All	Whole cell	179	Dissociated DRGs	(1) Cell size small 19–27 µm, medium 33–37 µm, large 44–54 µm (2) infliction point (3) Capsaicin + / −	Potassium currents	Unclear
[42]	All	Intracellular microelectrode	159	Intact DRG ex vivo	(1) CV and APD (2) Infliction + / −	RMP, threshold, APAmp, APD, AHP-duration, input resistance, AHP peak, infliction point, dep. slope, overshoot, firing pattern, size TTX, potassium currents	Post hoc
[17]	All	Whole cell	28	Dissociated DRGs	(1) Cell size small < 30 µm vs. large > 30 µm	Capsaicin, heat	Unclear
[23]	All	Intracellular microelectrode	97	Intact DRG ex vivo	(1) CV: C < 1.3 m/s; Aδ 2–12 m/s, Aα/Aβ > 12 m/s (2) SP + / −	RMP, APD, AHP-duration, AHP peak, infliction point, size, SP	Pre hoc
[18]	All	Whole cell	89	Dissociated DRGs	(1) Cell size small < 32.5 µm vs. large > 32.5 µm (2) heat sensitivity + / −	RMP, infliction point, size, capsaicin, heat	Pre hoc
[29, 30]	All	Whole cell	81	Dissociated DRGs	(1) P2x currents kinetics	Size, capsaicin, IB4	Unclear
[29]	Only 16–48 µm soma size	Whole cell	153	Dissociated DRGs	Internal clustering: current signature	APD, AHP duration, capsaicin	Post hoc
[5]	All	Intracellular microelectrode	104	In vivo DRG recordings	(1) Sensory testing and CV: C < 0.8 m/s; Aδ 1.4–6.5 m/s, Aα/Aβ > 6.5 m/s (2) Cell size small < 23 µm, medium 23–32 µm, large > 32 µm (3) Nav1.8 intensity	RMP, APD, RT, FT, overshoot, size, IB4	Pre hoc
[45]	Only 15–30 µm soma size	Whole cell	90	Dissociated DRGs	IB4 + / −	Threshold, APAmp, APD, AHP-duration, overshoot, TTXr, decay, TTP	Pre hoc
[13]	All	Whole cell	82	Dissociated DRGs	Cell size large > 35 µm vs. small < 30 µm	Capsaicin	Unclear
[22]	Small + medium DRGs	Whole cell	37	Dissociated DRGs	(1) Capsaicin sensitivity (2) IB4 + / −	pH response	Pre hoc
[6]	All	Intracellular microelectrode	120	In vivo DRG recordings	(1) IB4 + / − (2) CV (3) sensory testing	APD, AHP duration, capsaicin	Pre hoc
[39]	TTXr currents expressing DRGs	Whole cell	122	Dissociated DRGs	Cell size	TTXr	Unclear
[2]	All	Whole cell	47	Dissociated DRGs	IB4 + / −	RMP, threshold, AHP-duration, input resistance, overshoot, firing pattern, TTP, use-dependent inhibition, slow inactivation	Pre hoc
[15]	All	Intracellular microelectrode	50	Intact DRG ex vivo	CV + APD	RMP, threshold, APD, AHP-duration, input resistance, repetitive stimulation	Unclear
[36]	Only < 25 µm soma size	Whole cell	36	Dissociated DRGs	IB4 + / −	Repetitive stimulation, APD, threshold	Pre hoc
[49]	Cutaneous neurons	Whole cell + perforated patch	123	Dissociated DRGs	BKCa currents	Size, capsaicin, IB4	Unclear
[25]	All	Intracellular microelectrode	167	Intact DRG ex vivo	CV: C < 1.6 m/s; Aδ 2.9–8 m/s, Aα/Aβ > 14.3 m/s	RMP, APD, RT, FT	Unclear
[19]	Only 17.5–29 µm soma size	Whole cell	130	Dissociated DRGs	Potentiating of mechanically activated currents by low pH	IB4	Unclear
[33]	TRPM8 + DRGs	Whole cell	41	Dissociated DRGs	(1) TTXr vs. TTXs cells	RMP, threshold, APD, AHP-duration, AHP Peak, input resistance, TTXr	Unclear
[7]	All	Intracellular microelectrode	177	Intact DRG ex vivo	CV and infliction point	RMP, APAmp, APD, AHP-duration, repetitive stimulation, size	Unclear
[34]	Cutaneous neurons	Perforated patch	49	Dissociated DRGs	NCX activity + / −	Size, capsaicin, IB4	Pre hoc
[48]	Cutaneous neurons	Whole cell	141	Dissociated DRGs	Grouping via response on 300 µM nicotine: non responder, slow kinetic responder, fast kinetic responder	Size, capsaicin, IB4	Unclear
[4]	Only DRGs with CV < 0.8 m/s	Intracelluar microelectrode	78	In vivo DRG recordings	(1) Sensory testing	RMP, threshold, APD, AHP-duration, AHP peak, overshoot	Pre hoc
[41]	Only < 35 µm soma size	Whole cell	89	Dissociated DRGs	(1) Mechanical sensitivity (2) AP features: APD and infliction defining putative nociceptive DRGs	Threshold, APD, input resistance, firing pattern, size	Unclear
[46]	Only > 50 µm soma size	Whole cell	242	Intact DRG ex vivo	Internal clustering via: ramps, IF relationship, AP threshold	RMP	Post hoc
			3496

**Table 2:** Literature and extracted features human DRG. Summary of studies and extracted data for human DRGs. “Groups determination” indicates whether data were group post or pre hoc. *RMP*, resting membrane potential; *APD*, action potential duration; *capsaicin*, capsaicin sensitive; *TTXs/r*, tetrodotoxin resistant/sensitive; *dep slope*, depolarizing slope

	Cells included	Method	n Neurons	Preparation	Subgrouping into	Assessed parameters	Groups determination
[1]	All	Whole cell	40	Dissociated DRGs	Capsaicin + / −	APD, size,	Unclear
[3]	Small + medium DRGs	Whole cell	141	Dissociated DRGs	Shoulder size	APD, size, dep slope mean, max min	Unclear
[47]	All	Whole cell	226	Dissociated DRGs	TTXs/TTXr	Size	Unclear

All outcomes are narratively synthesized to provide an overview on each electrophysiological feature for different subgroups in DRGs. We chose this approach because of the large heterogeneity in the design of the included studies with respect to electrophysiological experimental approach (patch clamp vs. sharp electrode) and definition of AP analysis parameters (e.g., different analysis approaches in the determination of AP duration) and subgrouping within the studies which prevented a thorough meta-analysis.

Additionally, all outcomes assessed by at least two publications for comparable subgroups (e.g., resting membrane potential in dependence of CV) are presented in a semiquantitative analysis to assess overarching differences between DRG subgroups (see Table 3).

**Table 3:** Semiquantitative analysis of subgroup-outcome (highlighted in bold) combinations investigated by at least two studies. n study indicates number of studies for specific outcome subgroup combination, n cells/study indicates the number of cells included per study

	Group									
	CV–C-fibers vs. other		IB4 + vs. IB4 − DRGs		SP + vs. SP − DRGs		TTXs vs. TTXr current		Small vs. large DRGs	
**RMP**	**Depolarized**	**No effect**	**Depolarized**	**No effect**	**Depolarized**	**No effect**	**Depolarized**	**No effect**		
n study (n cells/study)	3 (*n* = 50 + 167 + 211 = 406)	2 (*n* = 70 + 159 = 229)	2 (*n* = 47 + 120 = 167)	0	1 (*n* = 246)	1 (*n* = 97)	0	3 (*n* = 41 + 78 + 242 = 361)		
**Input resistance**	**Higher**	**Lower**								
n study (n cells/study)	3 (*n* = 70 + 159 + 50 = 279)	0								
**AP threshold**	**Higher**	**Lower**	**Higher**	**Lower**						
n study (n cells/study)	0	3 (*n* = 211 + 50 + 159 = 420)	2 (*n* = 47 + 90 = 137)	0						
**AP amplitude**	**Higher**	**No effect**								
n study (n cells/study)	3 (*n* = 177 + 70 + 159 = 406)	1 (*n* = 211)								
**Overshoot**	**Larger**	**Smaller**	**Larger**	**No effect**						
n study (n cells/study)	3 (*n* = 70 + 159 + 211 = 440)	0	0	3 (*n* = 47 + 120 + 90 = 257)						
**APD**	**Longer**	**Shorter**	**Longer**	**Shorter**						
n study (n cells/study)	7 (*n* = 177 + 70 + 50 + 97 + 167 + 159 + 211 = 931)	0	2 (*n* = 90 + 120 = 210)	0						
**AHP duration**	**Longer**	**Shorter**	**Longer**	**No effect**						
n study (n cells/study)	3 (*n* = 70 + 159 + 211 = 440)	0	0	2 (*n* = 47 + 90 = 137)						
**AHP peak**	**Less hyperpolarized**	**No effect**								
n study (n cells/study)	2 (*n* = 211 + 159 = 370)	1 (*n* = 70)								
**Infliction point**	**Proportion higher**	**No effect**								
n study (n cells/study)	3 (*n* = 70 + 159 + 211 = 440)	0								
**Depolarization slope**	**Faster**	**No effect**								
n study (n cells/study)	2 (*n* = 70 + 159 = 229)	0								
**Rising time**	**Slower**	**No effect**								
n study (n cells/study)	2 (*n* = 211 + 167 = 378)	0								
**Falling time**	**Slower**	**No effect**								
n study (n cells/study)	2 (*n* = 211 + 167 = 378)	0								
**Time to peak**			**Longer**	**No effect**						
n study (n cells/study)			2 (*n* = 47 + 90 = 137)	0						
**Firing pattern**	**More single**	**More multiple**								
n study (n cells/study)	1 (*n* = 211)	1 (*n* = 159)								
**Cell size**	**Smaller**	**No effect**								
n study (n cells/study)	4 (*n* = 104 + 97 + 159 + 177 = 537)	0								
**Capsaicin sensitivity**									**Proportion higher**	**No effect**
n study (n cells/study)									4 (258 + 82 + 28 + 89 = 457)	0
**Heat sensitivity**									**Proportion higher**	**No effect**
n study (n cells/study)									2 (*n* = 28 + 89 = 117)	0

## Results

**Fig. 3:**
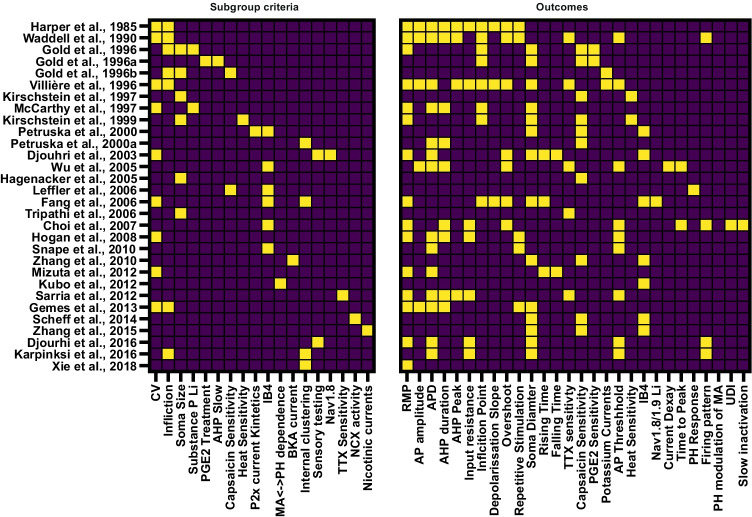
Rat DRG study subgroups and assessed AP parameters. Matrix showing the included publications using rat DRGs, which subcategorization was applied *(left)* and which outcome parameters were assessed in the mentioned studies *(right)*. LI, like immunoreactivity; MA, mechanically activated currents

In order to assess and distinguish electrophysiological features of DRG neuron subtypes, we were retrieving studies which compared at least two groups of sensory neurons (using a subgroup criterion, such as, e.g., CV) and collected the outcomes for these experiments (e.g., RMP or TTXr currents, Figs. 2 and 3). In a second step, we identified grouping criteria which were used in at least two studies to find electrophysiological characteristics which are likely to be commonly accepted among scientist to characterize specific subgroups (Table 3).

**Fig. 4:**
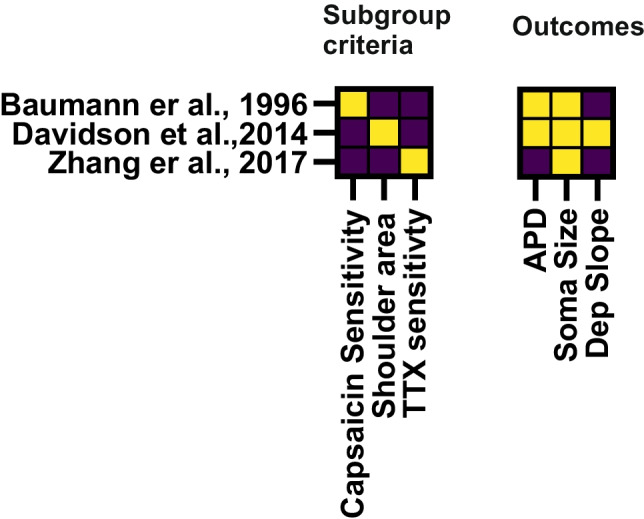
Human DRG study subgroups and assessed AP parameters. Matrix showing the included publications using human DRGs, which subcategorization was applied *(left)* and which outcome parameters were assessed in the mentioned studies *(right)*

The literature research initially identified 459 studies of which 30 studies on rat DRG tissue and three publications on human DRG tissue were incorporated (Tables 1 and 2). In the included studies, we found 18 criteria used to group rat DRGs (Fig. 3) and three for human DRG (Fig. 4). A total of 27 outcome parameters were extracted from the studies as illustrated in Fig. 2 and summarized in Fig. 3 for rat DRGs and Fig. 4 for human DRGs. The resulting data is summarized in Table 1 for rat DRGs and Table 2 for human DRGs.

The following criteria were used for subgrouping rat DRGs (Fig. 3, number in parentheses refers to number of studies using this criterion): CV (8), immune reactivity (8), the infliction point in the AP (7), the soma size (6), pharmacological approaches (5), isolation of specific currents/potentials in voltage/current clamp (4), sensory testing in the animal or internal clustering strategies (both 3), and mechanical or heat evoked currents in two studies (sum > 30 publications due to double categorization in some papers). The three studies on human DRGs used either capsaicin sensitivity, area of AP-shoulder, or ratio of TTXr/TTXs currents to form subgroups (Fig. 4).

As the data base for electrophysiological group distinctions in human DRG tissue is small, we emphasize that the conclusions drawn for possible interspecies similarities and distinctions shall not be taken without reservation until a broader pool of data is available.

In the following sections, each outcome of every parameter for DRG subpopulations is summarized. We start with results from current clamp and voltage clamp, then report on cell size, pharmacology, and immune reactivity. The outcomes of all included studies are compared with respect to the separation criteria in each study design.

### Rat DRGs

#### Resting membrane potential

15/30 included studies (50%) assessed RMP as a distinguishing parameter between DRG subgroups. Six of them used CV as grouping criterion and two of those reports found no significant differences [14, 42] while three state that C-fibers defined by CV display a more depolarized RMP [15, 25, 43]. When IB4 reactivity was used to separate cells, two studies congruently report a more depolarized RMP for IB4 negative DRGs [2, 6].

Using staining for substance P (SP) on the other hand showed a more depolarized RMP for SP positive DRGs in one study [8], while another found no significant differences [23]. The ratio of TTXr vs. TTXs does not seem to affect RMP: three publications concordantly reported no significant RMP changes with respect to TTXr vs. TTXs currents in (1) TRPM8 positive DRGs [33], (2) between LTM and HTM C-fibers [4] or (3) within a study defining subgroups in DRGs > 50 µm via AP threshold and ramp currents [46]. One publication compared Nav1.8 negative with positive DRGs and reported the latter to have a more depolarized RMP [5].

When subgrouping A-fibers into those with and without infliction points in the repolarizing phase of the AP, the RMP of A-fibers without infliction point was more depolarized than the RMP of C-fibers and A-fibers with infliction point [7]. DRGs showing an infliction point in the repolarizing phase were reported to have a more depolarized RMP [8] and heat sensitive DRGs were more depolarized compared to non-heat sensitive DRGs [18].

When considering these data, it seems likely that DRGs linked to rat C-fibers are more depolarized than other neurons, that IB4 + neurons are more hyperpolarized, and the ratio of TTXr to TTXs channels has no influence on RMP (Table 3).

#### Input resistance

5/30 (16.6%) of the included studies examined categorized DRGs for differences in input resistances. Not surprisingly, there is a clear distinction between fiber types as three of three publications reported higher input resistances for C vs. A fibers as defined by CV [14, 15, 42].

When comparing IB4 positive and negative cells, no significant change of input resistance was detected [2]. As with RMP, TTXs, and TTXr, current expressing neurons seem not to differ in their input resistance between in a subpopulation of Ca^2+^-imaging confirmed TRPM8 + cells [33].

In conclusion, it seems likely that DRGs linked to rat C-fibers have a higher input resistance than other neurons (Table 3).

#### Action potential threshold

9/30 (30.0%) of included studies assessed AP threshold as a distinguishing parameter between defined DRG subgroups. A lower threshold was identified for C vs. A fibers (assessed by CV) in three out of three publications [15, 42, 43], and one of them could even show a distinct threshold distribution for Aα/Aβ > Aδ > C fibers [42]. IB4 + DRGs were shown in two studies to have a higher AP threshold than IB4 − DRGs [2, 45].

The RMP at which cells have their minimal threshold for AP firing was more depolarized in IB4 − than IB4 + DRGs [36]. AP threshold was reported to be higher for TTXs vs. TTXr current expressing neurons in an included subpopulation of Ca^2+^-imaging confirmed TRPM8 + cells [33]. When including only CV characterized C-fibers, AP threshold of LTM fibers was shown to be lower than that of HTM fibers [4]. One study including neurons smaller than 35 µm defined DRGs to be putative nociceptive vs. non-nociceptive via AP duration and infliction point reported higher AP thresholds in the putative non-nociceptive group [41].

In summary, it seems likely that DRGs linked to rat C-fibers have a lower AP threshold than other neurons, and that IB4 + DRGs have higher AP threshold compared to IB4 − DRGs.

#### Action potential amplitude

5/30 (16.6%) of the included studies assessed DRGs for AP amplitude (APamp). Four of five studies subgrouped DRGs via CV, three of these four report C-fibers to have higher amplitudes than A-fibers [7, 14, 42], while one could not identify a significant APamp difference [43].

One of those publications subgrouped A-fibers into those with and without infliction points and showed that A-fibers with infliction points have higher amplitudes than those without [7]. Also IB4 expression does not seem to influence the AP height [45].

When considering these data, it seems likely that DRGs linked to rat C-fibers have a higher AP amplitude than other neurons.

#### Action potential overshoot

8/30 (26.6%) of the included studies examined categorized DRGs for their AP overshoot. Three of three publications reported the overshoot to be larger in C-fibers compared to A-fibers [14, 42, 43] with the exception that the first of those studies separated Aα and Aβ fibers and reported the overshoot relation to be is Aβ1 + C > Aα + Aδ + Aβ0 (β1: with infliction point, β0: no infliction point [14]).

Looking at all neurons independent from their fiber class, it was shown that cells with infliction point have a larger overshoot [43]. Similar to the findings for the AP amplitude, IB4 reactivity does not seem to affect the size of the AP overshoot, as was concordantly reported by three publications [2, 6, 45].

In an in vivo patch-clamp approach used to assess sensory qualities in anesthetized rats, the overshoot of nociceptive and stimulus unresponsive neurons was reported to be higher than that of LTM DRGs [5]. A similar follow up study by the same authors using the identical experimental approach confirmed those findings also for a subgroup of putative nociceptive C-fiber DRGs with a CV < 0.8 m/s [4].

When considering these data, it seems likely that DRGs linked to rat C-fibers have a larger overshoot than other neurons. IB4 reactivity seems to have no effect on the AP overshoot.

#### Action potential duration (AP duration)

15/30 included studies (50%) assessed AP duration as a distinguishing parameter between defined DRG subgroups. Seven of those 15 publications subgrouped DRGs via CV and all generally accord that C-fiber DRGs have a longer AP duration than A-fiber DRGs [7, 14, 15, 23, 25, 42, 43]. Small differences are reported concerning AP durations of A-fiber subgroups: one study reports Aδ-fiber related neurons to have longer AP duration than Aβ [14], in most other studies Aα and Aβ are pooled into one group. Five of seven publications state that the length of the AP duration graded as Aα/Aβ < Aδ < C fibers [7, 15, 23, 25, 43], 1/7 studies does not distinguish between A-fiber subtypes [42]. When an infliction point is present in the repolarizing AP phase, the AP duration is longer, as agreed upon by two studies [42, 43]. Two studies report AP duration to be longer in IB4 + DRGs [6, 45].

Heat sensitive DRGs display longer AP duration than those insensitive to high temperatures [18] and CV-defined A-fibers with more Nav1.8 immune reactivity were reported to have a longer AP duration [5]. In a subpopulation of Ca^2+^-imaging confirmed TRPM8 + cells, only TTXs expressing neurons had longer AP durations than those with TTXr currents [33]. One study including CV characterized C-fibers reported a shorter AP duration for LTM fibers than for HTM fibers [4].

In an approach to identify a functional clustering [29, 30], hyperpolarization activated currents as well as inward and outward conductances were used to identified a subset of small IB4 + DRGs with capsaicin sensitivity, slow ATP induced currents, and a small non-desensitizing response to low pH. This subgroup had a prolonged AP duration compared to the other in the study identified clusters [29, 30]. The sea anemone toxin ATX-II prolongs AP duration more strongly in IB4 − DRGs than in those positive for this leptin [36].

In summary, it seems likely that DRGs linked to rat C-fibers and IB4 + DRGs have a longer AP duration than other neurons.

#### Afterhyperpolarization duration

11/30 included studies (36.6%) assessed the duration of the AP afterhyperpolarization (AHP duration). Four of those eleven studies subgrouped DRGs via CV and three found the AHP duration of C-fibers to be longer than for A-fibers [14, 42, 43]. When focusing on A-fibers, Aα/Aβ showed a shorter AHP duration than Aδ- and C-fibers [15], although Aδ were reported earlier to have an even shorter AHP duration than Aα/Aβ (Aδ < Aα/Aβ < C) [14]. IB4 binding seems not to result in groups distinguishable by their AHP duration as shown in two independent studies [2, 45].

AHP duration of A-fibers with infliction point seems to be longer than that of A-fibers without [7] and similarly cells with TTXr currents in a subpopulation of Ca^2+^-imaging confirmed TRPM8 + cells display longer AHP duration than cells not expressing TTXr currents [33]. C-fibers identified by their CV showed shorter AHP duration for LTM fibers compared to HTM fibers [4]. The subgroup of small IB4 + DRGs with capsaicin sensitivity, slow ATP induced currents, and a small non-desensitizing response to low pH identified in Petruska et al. (29, 30 displayed a prolonged AHP duration compared to the other in the study identified by internal clustering analysis.

Thus, it seems likely that DRGs linked to rat C-fibers have a longer AHP duration than other neurons and that IB4 expression is not significant for this parameter.

#### Afterhyperpolarization peak

6/30 (20%) of the included studies assessed the peak of the AHP as a parameter for DRG subgroups. Of three studies categorizing DRGs via the CV, two show C-fibers to have a depolarized AHP peak compared to A-fibers [42, 43], of which the latter study also reports A-fibers with infliction point to be less hyperpolarized compared to those A-fibers without. On the other hand, one of the three publications which were using CV for categorization showed no significant difference for AHP peak between groups/fiber classes [14].

Aδ-fiber neurons with SP immune reactivity were shown to be more hyperpolarized than those negative for it [23]. C-fiber neurons were reported to have a more hyperpolarized AHP peak for LTM fibers compared to HTM fibers [4]. Only TTXs current expressing neurons compared to those with TTXr have a more hyperpolarized AHP peak in a subpopulation of Ca^2+^-imaging confirmed TRPM8 + cells [33].

Taken together, it seems likely that C-fibers have a depolarized AHP peak compared to other neurons.

#### Infliction point or “shoulder” of AP

8/30 (26.6%) of the included studies examined categorized DRGs for differences in the appearance of a so-called AP shoulder, i.e., an infliction point in the repolarizing phase of the AP defined by a second local minimum in its first derivative. Three studies analyzed infliction points in DRGs categorized by CV and all of them reported C-fiber DRGs to have infliction points in their APs [14, 42, 43]. One of those studies distinguished between Aα- and Aβ-fibers and reported more Aβ-fibers to have infliction points than Aδ-fibers (37.5% vs. 18%) [14]. The remaining two studies pooled Aα and Aβ fibers and reported the portion of cells with infliction points as Aα/Aβ < Aδ <  < C [42, 43].

Smaller DRGs are more likely to display an infliction point than larger neurons [8]. Cells displaying a Ca^2+^-dependent slow AHP were reported to have infliction points more frequently (Michael S. [8–10]. SP expression does not affect the number of cells with infliction point [23]. Heat sensitive [18] and IB4 + [6] DRGs are more likely to display a shoulder in their AP.

When considering these data, it seems likely that DRGs linked to rat C-fibers have more frequently infliction points than other neurons.

#### Maximum slope of AP upstroke

2/30 (6.6%) of the included studies examined categorized DRGs for differences of the maximum slope of the AP upstroke. Two found the slope in A-fiber DRGs to be faster than in C-fiber DRGs [14, 42], the latter one reported also the slope Aα/Aβ to be faster than Aδ.

When considering these data, it seems likely that DRGs linked to rat C-fibers have a faster maximum slope of the AP upstroke than other neurons.

#### AP rising time

4/30 ( 13.3%) of the included studies examined categorized DRGs for differences in the rising time (RT) of the AP, i.e., the time needed for the AP to reach its peak. Two of two studies reported the graded RT for fiber types as Aα/Aβ < Aδ < C [25, 43]. The latter one also reported DRGs with infliction point in the repolarizing phase to have a significantly longer rising time.

The AP rising time seems to correlate with the Nav1.8 immune reactivity for C nociceptive fibers, A-fibers, and A-fiber LTMs [5]. IB4 + DRGs were shown to have a longer rising time than IB4 − DRGs [6].

Thus, rat DRGs linked to C-fibers are likely to have a slower rising time than other neurons and IB4 + cells seem to have longer RT compared to IB4 − DRGs.

#### AP falling time

3/30 (10%) of the included studies examined categorized DRGs for differences in the falling time of the AP. Two of two studies reported the AP falling time for fiber types as Aα/Aβ < Aδ < C [25, 43] with the restriction that the latter one limits this statement to Aβ and Aδ fibers having an infliction point. This study also reports the FT of DRGs with infliction point to be slower compared to those without an infliction point. The AP falling time seems to correlate with the Nav1.8 immune reactivity for C nociceptive fibers, A-fibers, and A-fiber LTMs [5]. Fang et al. [6] reported a trend not reaching significance pointing towards a longer FT in IB4 + DRGs.

In summary, it is seems likely that DRGs linked to rat C-fibers have a slower falling time than other neurons.

#### Use-dependent inhibition and slow inactivation of voltage-gated sodium currents

1/30 studies (3.3%) showed that use-dependent inhibition of voltage-gated sodium currents was more pronounced in IB4 + vs. IB4 − DRGs [2]. This study also showed a significantly shorter time constant for entry into sodium channel slow inactivation in IB4 + vs. IB4 − DRGs [2].

#### Current decay and time to peak of voltage-gated sodium currents

2/30 studies (6.25%) compared time to peak (TTP) and current decay in DRG subgroups categorized by IB4 reactivity. Two studies showed a longer TTP in IB4 + DRGs [2, 45]. Choi et al. measure the time to AP peak in current clamp mode, while Wu and Pan report the time to sodium current peak in voltage clamp mode and also report a longer decay time constant current decay.

When considering these data, it seems likely that IB4 + DRGs have a longer time to peak.

#### Evoked APs by repetitive current stimulation

5/30 (16.6%) of the included studies examined categorized DRGs for differences in the response to repetitive current stimulation. One study reports that stimulating with increased frequency leads to an increase in AP duration only in C-fiber DRGs [14]. Contrary, another study describes a significant decrease in AP duration of C-fiber DRGs upon repetitive stimulation while the AP duration in A-fiber DRGs increased [7]. More specifically, C-fiber DRGs have a lower follow frequency than A-fiber DRGs, Aα and Aβ fibers without infliction point have faster following frequencies than Aβ-fibers with infliction point, and Aδ- and C-fiber DRGs and all A-fibers without infliction point have a faster following frequencies than A-fiber DRGs with infliction point [43].

When focusing on A-fibers, the following frequencies were Aα/Aβ > Aδ (no following frequencies for C-fiber DRGs reported) [15]. The action current amplitude (as defined as first derivation of AP multiplied with the negative cell capacitance) in response to repetitive stimulation with 2 Hz decreased more pronounced in IB4 + DRGs compared to IB4 − [36].

#### Firing pattern

4/30 (13.3%) of the included studies examined categorized DRGs for their firing pattern responses upon current injection.

The proportion of single vs. multiple AP firing is reported to be higher in A-fibers compared to C-fibers [43]. In contrast to that, another study reports the proportion of single AP firing neurons to be Aα/Aβ < Aδ < C [42].

The number of elicited APs upon current injection is described to be higher in IB4 − cells compared to IB4 + cells [2]. Small putative nociceptive neurons (as defined by the author by existence of an infliction point and long AP duration) < 35 µm may have more often multiple APs as current response than those categorized as non-nociceptive [41].

#### Voltage-gated potassium currents

2/30 (6.6%) investigated DRG subgroups for differences in their voltage-gated potassium currents.

A study describing 6 different potassium currents in DRGs characterizes a transient high threshold “I_AHT_” current to be selectively present in small, a transient rapid “I_AF_” current to be more present in large vs. small DRGs and DRGs without infliction point, and a sustained potassium current “I_ki_” to be more present in DRGs with infliction point [9]. When comparing time-dependent hyperpolarization activated currents (I_h_ current), no significant changes in subgroups defined by CV were identified [42].

#### Cell size

14/30 (46.6%) of the included studies investigated subgrouped DRG populations for changes in cell size. Four of four publications reported the cell size of C-fiber neurons to be smaller than cell size of A-fiber neurons [5, 7, 23, 42], and three of those studies conclude that the size relation was Aα/Aβ > Aδ > C [5, 23, 42].

Nav1.8 positive cells were found to be smaller than Nav1.8 negative cells [5]. Fast P2x currents occur only in small, mixed P2x current in small to medium DRGs, and slow P2x current only in medium-sized DRGs [29, 30]. No significant difference in median cell size was detected between heat sensitive and non-heat sensitive DRGs in the defined subset of small diameter DRGs [18]. SP positive cells seem to be smaller than those not expressing SP [8].

A Ca^2+^-dependent slow AHP seem to be linked to a small cell size [8–10] as well as IB4 + reactivity [6]. Small and large DRGs appear to have more voltage-dependent Ca^2+^ modulated K^+^ currents than medium DRGs [49]. The activity of sodium-calcium exchanger (NCX) can be found more often in small DRGs [34] and the kinetics of their nicotine evoked currents is slower compared to those in medium-sized DRGs [48]. The small putative-nociceptive neurons categorized via AP duration and existence of an infliction point were reported to be larger than the neurons classified as non-nociceptive [41]. Also use-dependent inhibition of voltage-gated sodium currents seems to be stronger in small DRGs which express TTXr [39].

When considering these data, it seems likely that DRGs linked to rat C-fibers are smaller than other neurons.

#### TTXr and TTXs voltage-gated sodium currents

3/30 (10%) of the included studies investigated subgrouped DRG populations for changes TTX response of their voltage-gated sodium currents. The fraction of cells showing TTXr currents is significantly higher in DRGs whose APs have infliction points [43].

The size of TTXs current was smallest in Aα/Aβ fiber DRGs, medium in Aδ, and largest in C-fiber DRGs [42]. One study reports the current density of TTXr to be higher in IB4 + vs. IB4 − DRGs [45].

#### Capsaicin

10/30 (33.3%) of the included studies investigated subgrouped DRG populations for changes in their response to capsaicin. Four studies showed that the average cell size of capsaicin-sensitive cells is smaller than the average cell size of capsaicin-insensitive cells (M. S. [8, 13, 17, 18].

The number of DRGs with a Ca^2+^-dependent slow AHP is higher in capsaicin-sensitive DRGs [8–10] and also heat sensitive cells are more numerous in capsaicin-sensitive cells compared to those non-responsive to this chemical [18]. Ninety percent of fast P2x currents expressing neurons, 33% of slow P2x expressing currents, and 25% of mixed P2x-expressing currents are capsaicin sensitive [29, 30]. The same study reported 100% of IB4 + cells to be capsaicin responsive.

The functionally categorized cells by Petruska et al. showed significant differences in their capsaicin response: small DRGS with fast ATP reacting currents, a non-desensitizing pH response, IB4 + , a long AP duration, and prolonged AHP showed amplified capsaicin reaction than the other clusters [29, 30]. DRGs expressing Ca^2+^ modulated K^+^ currents do not differ in their capsaicin sensitivity [49]. No difference in capsaicin sensitivity was observed between DRGs with different kinetics of nicotine evoked currents [48]. One study reports capsaicin sensitivity to be more frequent in DRGs with activity of the sodium-calcium exchanger (NCX) [34].

When considering these data, it seems likely that DRGs with smaller soma diameter are more frequently capsaicin sensitive.

#### Heat sensitivity

2/30 (6.6%) investigated DRG subgroups for differences in heat sensitivity. Both report consistently small DRGs to be heat sensitive more frequently than large DRGs [17, 18].

Thus, DRGs with smaller soma diameter are more frequently heat sensitive.

#### PGE2 and pH response

1/30 (3.3%) of the studies investigated PGE2 sensitization as a distinguishing parameter in DRG subgroups and showed the effect of PGE2 sensitization to be more pronounced in small DRGs as compared to larger ones [8]. 1/30 studies (3.3%) showed that inward currents in response to low pH was more frequent in both capsaicin sensitive vs. capsaicin insensitive and IB4 − vs. IB4 + DRGs. This study included only small- and medium-sized DRGs [22].

#### Substance P

1/30 (3.3%) investigated subgrouped DRGs for differences in SP immune reactivity and reported SP + cells only in Aδ- and C-fiber DRGs characterized by CV [23].

#### IB4 labelling

6/30 (20%) of the included studies investigated DRG subgroups for IB4 reactivity.

IB4 + neurons can be found with increasing numbers in Aα/Aβ < Aδ < C fiber neurons and more frequently in nociceptive and unresponsive (defined by the author as DRGs not responding to mechanical or thermal stimulation) C-fiber DRGs compared to LTM C-fiber DRGs [6]. More IB4 signal was described in DRGs with voltage-dependent Ca^2+^ modulated K^+^ currents compared to DRGs lacking this specific current [49]. The pH-dependent modulation of mechanically evoked currents seems to be more pronounced in IB4 + cells [19]. Slow nicotine evoked currents occur more frequently in IB4 + DRGs and fast nicotine evoked currents more frequently in IB4 − DRGs [48]. DRGs with activity of the sodium-calcium exchanger (NCX) are reported to be IB4 + [34]. One study reports the fraction of P2X currents in IB4 + fast P2x currents > mixed P2x currents > slow P2x currents > no P2x currents [29, 30].

#### Nav1.8/1.9 intensity

2/30 studies (6.6%) investigated DRG subgroups with regard to their Nav1.8 or 1.9 immune reactivity. In one of those studies, more Nav1.8 signal was reported in nociceptive and unresponsive than LTM fiber DRGs and more Nav1.8 signal was seen in Aδ- and C-fiber DRGs than in Aα/Aβ fiber related DRGs in in vivo recordings [5]. Another study reported more Nav1.9 expression in IB4 + vs. IB4 − DRGs [6].

### Human DRGs

#### Cell size

2/3 included studies (66.6%) assessed human DRG subgroups with respect to cell size. The cell diameter of capsaicin responsive cells seems to be smaller than that of unresponsive neurons [1]. The TTXr and TTXs ratio on the other hand was comparably distributed between all cell sizes in human dissociated DRG neurons [47].

#### Action potential duration

2/3 included studies (66.6%) assessed human DRG subgroups with respect to AP duration.

One publication found the AP to be longer in capsaicin responsive cells [1]. The area of the AP shoulder in the repolarizing phase positively correlates with AP duration [3].

#### Slopes

1/3 included studies (33.3%) assessed human DRG subgroups with respect to slopes. This study described no dependency between AP shoulder area in the repolarizing phase of the AP and the mean depolarizing slope or the maximum or minimum depolarizing slope [3].

### Semiquantitative meta-analysis

Taking together all studies investigated in this review, it becomes clear that there are five grouping criteria which were used often by neuroscientists to do a functional classification of DRGs: Fiber class as determined by CV, IB4 or SP staining, expression of TTXs/TTXr currents, and cell size. In our semiquantitative meta-analysis, we focused on parameters which were determined within these groups by at least two studies (Table 3). From this data, we can state for rat DRGs that: (1) C-fibers compared to other fibers are more depolarized, have a higher input resistance, AP threshold, AP amplitude, overshoot, a longer AP and AHP duration, a more depolarized AHP peak, more cells display infliction points, and their subthreshold depolarization is quicker, while the rise time and fall time of the AP is slower. Their firing pattern is rather tonic and cells are smaller. (2) IB4 expression is linked to a more depolarized RMP, a higher AP threshold, and longer AP duration as well as time to AP peak. It does not seem to affect overshoot or AHP duration. (3) The expression of TTXr currents does not affect RMP. (4) Small cells are more likely to be capsaicin and heat sensitive than larger neurons.

For human DRGs, there are currently only three studies published which categorize the cells, thus the data basis is much less solid. Still the studies showed, comparably to rat DRGs, that smaller human DRGs are more sensitive to capsaicin [1], and that capsaicin reactive cells have a longer AP duration [3] although the latter study did not include large diameter DRGs. One open research question remains if nociceptive human DRGs are more frequently smaller in size, as this is the case for rodent sensory neurons. TTXr current as an indicator for nociceptive neurons was not related to smaller diameter neurons [47], while in rat DRGs, TTXr currents are connected to C-fiber DRGs [42].

We put these data together with the intention to ease comparison to human sensory neurons and to help to improve translation where species differences may become obvious.

## Discussion

This systematic review merges the knowledge on electrophysiological approaches to identify subgroups within sensory neurons of rats and humans. Thirty studies for rat and 3 studies for human DRGs were included.

The presented data shows that there is broad knowledge on the electrophysiology of subgroups in rat DRG tissue that is, if studied in a comparable manner, frequently congruent. On the other hand, for human DRG tissue, the availability of data is far more restricted. Being aware of the very limited database for profound conclusions, it seems that there are both similarities and differences between the two species. Capsaicin sensitivity seems to be associated with broader action potentials and to be restricted to small diameter neurons in both species. On the other hand, considering the accuracy of translating the findings from rat DRG on human DRG tissue, the published data suggests that subgroups in sensory neurons of the two species show different electrophysiological behavior. Extended pharmacological characterization in direct comparison between human and rat DRGs revealed differences [47] and consistently reported electrophysiological outcomes in rat DRGs with respect to cell size and infliction points [8, 43] could at least partly not be verified in human DRG tissue [3, 47]. Axon length and cell size in human DRGs exceeds rat DRGs by far which implicates different physical and physiological challenges for those functional units [12]. Lastly, not all possible categorization approaches used in rodents are applicable to human DRGs. IB4 labelling has been used extensively to distinguish subgroups of rodent DRGs while human primary sensory neurons do not bind IB4 [35]. Very recently, single cell transcriptome data has become available for human and non-human primate DRG tissue [20, 26, 37]. In congruence with partly overlapping and distinct functional findings pointed out in this study, comparison of those data sets with rodent data sets revealed both overlapping and distinct features in the somato-sensation of both species. For example, classic TRPM8 positive cold sensing neurons seem to be further subdivided into two neuronal subgroups in humans, of which one is additionally expressing SCN10A, SCN11A, and PIEZO2, therefore likely rendering it sensitive to light touch [26]. Also, the accepted distinction between peptidergic and non-peptidergic nociceptors in rodents seems to be less clear in human sensory neurons [26, 37]. Those differences in transcriptomic organization most likely will affect the functional properties of neurons and can explain the functional distinctions described between the species. Bearing in mind the very limited available data on human tissue, further research is needed and will help to better understand the linkage between genetic identity, function, and evolution of somato-sensation.

### Categorization of DRG by electrophysiology

Sensory neurons are identified as a heterogeneous population since a long time and the first included paper in this review was published 1985. Nevertheless, electrophysiological characterization of DRG neuron subtypes still has and even gains new relevance for a couple of reasons: (1) The increasing knowledge on functional categorization of nociceptors with microneurography gives new perspectives on the diversity of human nociceptive neuronal functional units [24], (2) identification of mechanisms in which individual subtypes are especially involved into pathological phenotypes underlines the importance of understanding the molecular machinery behind neuronal subpopulations; (3) recent breakthroughs in single cell sequencing methods offer the possibility to categorize DRGs on a molecular expression level with unprecedented detail [20, 40] and combination of functional and expressional data in PatchSeq experiments allows for a deeper understanding of the molecular machinery underlying specific sensory neuron function [28]. (4) Large setbacks in the development of new analgesic treatments have shown limitations of the translation of rodent models to the human nociceptive system [16, 32] and thus there is growing consensus that assessing biological systems closer to the human are promising approaches in the development of better pain treatments. This involves on the one hand primary neuronal cultures of human neuronal tissue or species closer related to humans than rodents [20, 31]. On the other hand, also the differentiation of induced pluripotent stem cells (iPS-cells) into human nociceptors is a promising approach [21], especially as the access to and the availability of human DRGs is very elaborate and limited.

Beside electrophysiological characterization, sensory neurons have also been studied with more holistic imaging approaches offering the possibility to investigate also neuron population level of information encoding. As grouping parameters and readouts of imaging studies are only partly overlapping, a correlation with those study types is inherently imitated. Nevertheless, these studies show overlaps and affirmation of the data presented by the included patch-clamp data. For example, in the study of Teichert et al. [38], in which more than 2000 sensory neurons where exposed to various challenging compounds and assessed via calcium imaging, the authors show that capsaicin and ATP responsive cells seem to have a smaller diameters then ACH only responsive neurons which is in congruence with the shown patch-clamp results. Also the study of Wang et al. [44] using in vivo recordings of sensory shows that heat sensitivity is more frequently appearing in small diameter DRGs which is in congruence with the electrophysiological data included in this study.

The presented systematic review emphasizes that electrophysiological data on human or human-like sensory neurons is fragmented and further research is needed. During the examination of literature that match the inclusion criteria, we recognized that a large fraction of the available studies does not provide raw data access. With perspective on the modern view on data transparency and in the course of development of data mining possibilities, we emphasize the additional benefit of providing those resources to the research community.

### Limitations

Due to the heterogeneity of the included studies (see also Fig. 3), a quantitative analysis of the included studies did not seem appropriate. The assessed subgroups in the included publications differ substantially. Even if the same concepts were applied to classify DRGs, the parameterization differed, e.g., the definition of a “small DRG” differed by several µm or cutoff values between conduction velocities of different fiber types were not uniformly chosen by the investigators. This was partly due to the fact that also the tissue preparation was heterogeneous in the included study, e.g., dissociated DRGs vs. in situ patch-clamp approaches. Additionally many included studies did not clearly point out whether the investigated subgroups were defined pre hoc or post hoc, which would have increased the validity of the presented data. On the other hand, the include studies lead to a broad data set of 3903 included neurons (3496 rat DRGs and 407 human DRGs). It is still possible that our PUBMED search, although revealing a relatively comprehensive set of publications, did not catch all important papers. The procedure of using a systematic literature research and data extraction in combination with a narrative data synthesis offered to include a broad spectrum of suitable literature and to at least partly overcome the limitations emerging from the heterogeneity of the included studies.

## Conclusion

In conclusion, this study provides a detailed synthesis of the available electrophysiological data characterizing subtypes in primary sensory neurons in rats and humans. We highlight incompleteness in the electrophysiological description of human DRG neurons and elaborate that findings from rat neurons cannot be transferred offhand to the human system. Further functional data on human sensory neurons is needed, ideally in combination with genetic information on single cell scale to broaden our understanding of the nociceptive system in humans and to offer urgently needed progress in the development of analgesic treatment options.

## Declarations

### Study registration

The study was not registered beforehand.

## Abbreviations

AHP: Afterhyperpolarization
AP: Action potential
APamp: Action potential amplitude
APD: Action potential duration
CV: Conduction velocity
Dep: Depolarizing
DRG: Dorsal root ganglion
FT: Falling time
IB4: Isolectin binding protein 4
HTM: High-threshold-mechanoreceptive
LTM: Low-threshold-mechanoreceptive
RT: Rising time
RMP: Resting membrane potential
SI: Slow inactivation
SP: Substance P
TG: Trigeminal ganglion
TRPM8: Transient receptor potential cation channel subfamily M (melastatin) member 8
TTP: Time to peak
TTXr/s: Tetrodotoxin resistant/sensitive
